# GAD65 Positive Autoimmune Limbic Encephalitis: A Case Report and Review of Literature

**DOI:** 10.4021/jocmr1080w

**Published:** 2012-11-11

**Authors:** Abhishek Sharma, Divyanshu Dubey, Anshudha Sawhney, Kalyana Janga

**Affiliations:** a Department of Medicine, Maimonides Medical Center, New York, USA; b Department of Neurology, UT Southwestern, Texas, USA; c Department of Medicine, Pt. Madan Mohan Malviya Hospital, New Delhi, India

**Keywords:** Autoimmune, Limbic encephalitis, GAD Antibody, Seizure

## Abstract

Limbic encephalitis is a rare disorder affecting the medial temporal lobe of the brain, sometimes also involving hippocampus atrophy. It was initially considered to be only of paraneoplastic origin but now auto-immune (non-paraneoplastic) cases have also been reported. Most common non paraneoplastic antibodies associated with limbic encephalitis are Voltage gated potassium channel antibodies, NMDA receptor antibodies and GAD receptor antibodies. We present a case of limbic encephalitis which presented with sudden onset seizures which was preceded by confusion, disorientation and other psychiatric symptoms for a period of 5 weeks. No tumor was found on imaging and the classic paraneoplastic panel was negative. CSF and serum examination showed high titers GAD65 antibody guiding towards a diagnosis of non paraneoplastic limbic encephalitis. Her symptoms and GAD 65 antibody titers showed significant improvement following immunomodulatory therapy. The case presented here is unique and scientifically relevant, as it intends to raise awareness of Auto-immune Limbic Encephalitis, a potentially reversible cause of a medical emergency.

## Introduction

The field of medicine is filled with multiple challenges. Few of the challenges are related to the diagnosis of the underlying condition, others pertaining to restricted therapeutic options. Limbic encephalitis (LE) as a pathology poses both these challenges. Myriad of clinical presentation and lack of symptom specificity including seizure, memory problems, irritability, depression, confusion and dementia leads to a wide differential diagnosis [1]. Viral infections, inflammatory or autoimmune disorders (lupus, Sjogren’s, Hashimoto thyroiditis and CNS vasculitis), toxic and metabolic encephalopathies, and paraneoplastic syndromes become the possible clinical etiologies.

Limbic encephalitis is a rare disorder affecting the medial temporal lobe of the brain, sometimes also involving hippocampus atrophy. It was first described by Breirly et al in 1960s when they reported 3 cases of sub-acute encephalitis involving the limbic area [2]. In 1968 Corsellis et al coined the term “limbic encephalitis” and also established the relationship between limbic encephalitis and systemic cancer [3]. Inflammatory involvement of the limbic area of the brain can also be explained by infective (viral) and systemic autoimmune (Sjogren's syndrome, systemic lupus erythmatosus, hashimoto thyroid it's, sarcoidosis) etiologies. Many neuronal antibodies have been associated with LE. These can be directed either against intracellular (classic paraneoplastic) antigens, including Hu, CV2/CRMP5, Ma2 and amphiphysin or against cell membrane antigens, including voltage-gated potassium channels, N-methyl-D-aspartate receptor and Glutamic Acid Decarboxylase receptors expressed in the neuropil of hippocampus and cerebellum [4, 5]. The former category is related to cancer (paraneoplastic limbic encephalitis), showing limited response to immunomodulatory therapy whereas the latter category is less frequently associated with tumors and responds significantly better to immunomodulatory therapy. It has also been recognized that some patients presenting with limbic encephalitis with negative antibody screen in serum and CSF show full recovery after treatment with steroids or immunomodulatory therapy indicating an autoimmune etiology [6, 7]. We present a case of a patient with rapidly deteriorating cognitive function and recurrent seizures. She was diagnosed with autoimmune limbic encephalitis associated with GAD65 antibodies. Her symptoms showed dramatic improvement following immunomodulatory therapy. The case presented here is unique and scientifically relevant, as it intends to raise awareness of Auto-immune Limbic Encephalitis, a potentially reversible cause of a medical emergency.

## Case Report

A 22-year-old right handed woman without any significant past medical history presented with episodes of sudden onset seizures, inability to recognize immediate family members and familiar places, apathy, bizarre behaviors, and brief episodes of disorientation for 5 weeks. On examination she was scored 24/30 on Mini-mental state examination. Cranial nerve, language, sensory, motor and cerebellar function test were within normal limits. MRI showed bilateral signal hyper intensity in temporal lobes.

Lumbar puncture was performed and CSF analysis showed no pleocystosis and CSF protein and glucose were within normal limit. PCR for HSV and HHV-6 was negative. Seizure episodes were documented on video-EEG monitoring. Psychiatry expert were also consulted which ruled out any psychiatric disorder for patient symptoms. A diagnostic search for cancer including imaging and serological studies for various tumor markers was negative. Evaluation of typical antibodies (Hu, CV2/CRMP5 and Ma2 etc.) present in a paraneoplastic panel also turned out to be negative. Autoantibody markers in CSF and serum showed presence of presence of GAD65 autoantibody, but evaluation of NMDA receptor antibodies and Voltage gated potassium channel antibody (VGKc) was negative. Suspecting an autoimmune etiology immunomodulatory treatment with intravenous immunoglobulin and intravenous methyl-prednisone was initiated, which subsequently resulted in marked improvement in symptoms. Subsequent assessment of GAD65, post-immunomodulatory therapy showed a decrease in titers of GAD65 antibody.

## Discussion

Limbic encephalitic syndrome presents as a diagnostic challenge. Classic presentation includes the rapid development of irritability, short-term memory loss, depression, sleep disturbances, hallucinations and seizures. Confusion, repetition of same questions over and over, staring episodes and involvement of temporal lobe is hallmark of LE patients. Autobiographical memory is characteristically preserved in this disorder. LE usually has a sub-acute development, in days or weeks, presenting initially as short-term memory deficits, but this deficit is surprisingly overlooked in some patients [1].

Initial investigation in these patient commonly include CT of the brain with contrast, LP with cell count, protein, glucose (and serum glucose), viral screen (especially HSV, HHV-6), acid-alcohol fast bacilli (AAFB) smear, bacterial and mycobacterium culture. CSF and serum examination for anti-neuronal antibodies, paraneoplastic proteins and antibodies against neuronal cell membrane antigen is also essential. An MR brain is mandatory, in case of LE fluid-attenuated inversion recovery (FLAIR) or T2 sequences could show hyper intense signal in the medial aspect of the temporal lobes. These abnormalities are often asymmetric, and sometimes seem to be unilateral [6, 8]. Contrast enhancement is infrequent, but can occur with all clinical-immunologic variants of limbic encephalitis [9]. Sometimes clinically classic limbic encephalitis may also have a normal MRI, hence manifesting as a diagnostic challenge. Electro-encephalography shows epileptiform activity in either one or both temporal lobes. It may also show focal or generalized slowing in the temporal areas [8].

There are two sets of diagnostic criteria utilized for diagnosis of paraneoplastic limbic encephalitis. First criterion was proposed by Gultekin et al in 2000 (Table 1), which was later revised by Graus et al in 2005 (Table 2) [10, 11].

**Table 1:** Gultekin Diagnostic Criteria for Limbic Encephalitis

Either, Histopathological evidence of limbic encephalitis
OR, Presence of following:
-Psychiatric symptoms ,short-term memory loss, or temporal lobe seizures suggestive of limbic system involvement
-Time period between onset of neurological symptoms and cancer diagnosis < 4 years.
-Exclusion of other etiologies causing limbic encephalopathy : infection, metastases, metabolic causes, stroke and medication side-effects
-At least one of
-Inflammatory findings in CSF
-MRI FLAIR or T2 showing bilateral hyper intensity
-EEG showing focal temporal lobe changes either epileptic discharges or slowing

**Table 2:** Graus and Saiz Diagnostic Criteria for Limbic Encephalitis

-Sub-acute onset (few days to 12 weeks) of short-term memory loss, seizures, confusion and psychiatric symptoms and
-Evidence of limbic system involvement (Radiologic or Neuropathologic) and
-Time period between onset of neurological symptoms and cancer diagnosis < 5 years or development of limbic dysfunction symptomatology in association with a well-defined paraneoplastic antibody (amphiphysin, CV2, Hu, Ma2, Ri) and
-Exclusion of other etiologies explaining above symptoms

Forty to fifty percent of patients with limbic encephalitis do not have any of the classic paraneoplastic or antineuronal antibodies [7].Therefore 2 broad categories of autoimmune limbic encephalitis were formulated: (1) one associated with antibodies to intracellular neuronal antigens, which include all previously known paraneoplastic antigens, and (2) one associated with antibodies to cell membrane antigens, including the VGKc, NMDA receptor, GAD [12].

The above case describes a young lady with anti-GAD positive limbic encephalitis who responded well to immune-modulatory therapy and the GAD antibody level decreased after the therapy. Inspite of thorough examination and investigation no underlying tumor, systemic autoimmune etiology (like SLE, hasimoto thyroiditis, Sjogren’s syndrome) or infection was found. Decrease of GAD antibody titers with therapy along with improvement of symptoms, points toward the possible causal relationship of GAD65 antibody with limbic dysfunction.

Association between GAD antibody and non-paraneoplastic LE was shown by Mata et al [13]. These antibodies have been associated with some other neurological disorders as well such as stiff-person syndrome, cerebellar ataxia and palatal myclonus. They are also seen in patients with anti GABA (B) receptor encephalitis which is frequently associated with Small cell lung cancer or neuroendocrine tumor of lung [14]. GAD catalyzes production of gamma-aminobutyric acid (GABA). Exposure of these intracellular antigens during exocytosis could lead to formation of these pathologic antibodies [15, 16]. It is proposed that these antibodies subsequently reduce the synthesis of GABA or interferes with exocytosis of GABA in the nervous system [17]. A study conducted by Malter et al showed Anti GAD antibody positive limbic encephalitis cases presented at a younger age compared to VGKc antibody encephalitis. These cases are more commonly found in females than males. Common initial presentation is seizure, which remains resistant to anticonvulsive therapy [18]. Other studies have shown patients with GAD antibody LE presenting initially with short term memory loss [19].

Corticosteroids, intravenous immunoglobulin and plasma exchange are most frequently used as therapeutic options. Other immune-suppressive agents like cyclophosphamide and rituximab can also be utilized as a therapeutic option [7, 20]. GAD antibody LE patients are far more resistant to methyl prednisone treatment compared to VGKc antibody LE patients. But these patients have responded well to intravenous immunoglobulin and plasma exchange [13, 21]. A study conducted by Saidha et al also shows promising results with decrease in seizure frequency and improvement in Behavior memory testing in these patients with use of mycophenolate mofetil [22]. Development of these therapeutic options highlights the need for early detection and aggressive management for patients with autoimmune limbic encephalitis. If this disease process is considered early, diagnosed promptly and treated appropriately, it can be reversed and the patient restored to their premorbid state.

### Conclusion

Limbic encephalitis, when initially discovered in 1960s was believed to be exclusively of paraneoplastic origin, but there have been an increasing frequency of cases in adults and pediatric population without evidence of a tumor at presentation. Anti-GAD receptor antibody-positive limbic encephalitis is an emerging diagnosis amongst the adult population. It is important that we consider autoimmune limbic encephalitis in our differential diagnosis in adults with encephalopathy, particularly if psychiatric symptoms are seen. Therapeutic responsiveness of this condition reiterates the importance of diagnosing a reversible neurologic pathology. With timely intervention, clinicians may be able to avoid permanent cognitive and behavioral damage.
